# Hemolytic Crisis following Naphthalene Mothball Ingestion in a 21-Month-Old Patient with Glucose-6-Phosphate Dehydrogenase (G6PD) Deficiency

**DOI:** 10.1155/2019/1092575

**Published:** 2019-06-19

**Authors:** Maricel Dela Cruz, Muhammad Masood Khalid, Ahmed Mostafa, Muhammed Ershad, David Vearrier, Rita McKeever

**Affiliations:** Department of Emergency Medicine, Division of Medical Toxicology, Drexel University College of Medicine, 245 N 15 Street, MS 1011, Philadelphia, PA 19102, USA

## Abstract

**Introduction:**

Naphthalene is an aromatic hydrocarbon that may be found in mothballs and deodorizers. Exposure can occur by ingestion or dermal absorption. We present a case of acute hemolysis requiring blood transfusion in a 21-month-old male with a history of glucose-6-phosphate dehydrogenase (G6PD) deficiency after ingestion of a naphthalene-containing mothball.

**Case Presentation:**

A 21-month-old male with G6PD deficiency presented to the emergency department three hours following an exploratory ingestion of a naphthalene-containing mothball. On arrival, the patient was tachycardic with normal blood pressure, temperature, respiratory rate, and oxygen saturation. Initial laboratory studies showed significant anemia with elevated reticulocyte count, blood urea nitrogen, total bilirubin, and lactate dehydrogenase. Haptoglobin level was low, and the methemoglobin level was unremarkable. The patient was admitted to the pediatric intensive care unit and underwent blood transfusion.

**Discussion:**

This case serves as a reminder that mothballs, a ubiquitous household item, can be hazardous when accessible to vulnerable children. Care should be taken to secure these products and prevent ingestion.

## 1. Introduction

Naphthalene is an aromatic hydrocarbon that may be found in mothballs, deodorizers, or insecticides [1, 2]. Ingestion of naphthalene-containing products may potentially produce methemoglobinemia or hemolysis. This is especially true in patients particularly susceptible to these processes, such as individuals diagnosed with glucose-6-phosphate dehydrogenase (G6PD) deficiency [3, 4]. We present a case of acute hemolysis requiring blood transfusion in a 21-month-old male with a history of G6PD deficiency after ingestion of approximately half of one naphthalene-containing mothball.

## 2. Case Presentation

A 21-month-old male presented to the emergency department (ED) three hours following an exploratory ingestion of half of a naphthalene-containing mothball. Vital signs on arrival were heart rate 163 beats per minute, blood pressure 99/55 mmHg, temperature 99.4 degrees Fahrenheit, respiratory rate 44 breaths per minute, and oxygen saturation 95% on room air. The patient did not initially present with gastrointestinal symptoms prior to arrival but in the ED exhibited nonbilious, nonbloody vomiting, with four subsequent episodes overnight. Physical examination was otherwise unremarkable with no signs of jaundice or abdominal tenderness. The patient was given one 20 mL/kg bolus of normal saline and 2 mg of sublingual ondansetron to manage his tachycardia, tachypnea, and vomiting.

Initial laboratory studies were remarkable for a hemoglobin of 4.5 g/dL (reference 9.6 to 15.6 g/dL), hematocrit of 14.4% (reference 34.0 to 48.0%), reticulocyte count of 6.8% (reference 0.5 to 1.5%), blood urea nitrogen of 22 mg/dL (reference 4 to 13 mg/dL), total bilirubin of 4.06 mg/dL (reference 0.00 to 1.00 mg/dL), lactate dehydrogenase (LDH) of 886 units/L (reference 155 to 345 units/L), haptoglobin of less than 15 mg/dL (reference 33 to 188 mg/dL), and methemoglobin of 1.8% (reference 0.0 to 1.4%). Creatinine was found to be less than normal at 0.23 mg/dL (reference 0.3 to 0.7 mg/dL). Chest and abdominal radiographs were negative for radiopaque foreign bodies. Abdominal ultrasound was not performed. Red blood cell morphology included microcytosis, hypochromasia, and polychromasia. Direct antiglobulin testing for IgG and C3 was both negative.

Review of the patient's outpatient medical records revealed that he was diagnosed with G6PD deficiency at 13 months of age. A hemoglobin level performed as an outpatient a year prior to this visit was 11.7 g/dL. Due to the ingestion of a naphthalene-containing mothball, concerning the signs of acute hemolysis, the decision to transfuse the patient with packed red blood cells was made.

After successful transfusion of two units of 7.5 ml/kg packed red blood cells four hours later, the patient's vital signs improved. Repeat laboratory findings at that time included a hemoglobin of 7.7 g/dL, hematocrit of 22.6%, reticulocyte count of 0.9%, blood urea nitrogen of less than 5 mg/dL, total bilirubin of 0.44 mg/dL, LDH of 359 units/L, and methemoglobin of 0.5%.

The patient was discharged after 72 hours of continuous observation, and repeat trending of the patient's complete blood count showed a normal CBC, reticulocyte count, LDH, and comprehensive metabolic panel. The patient remained asymptomatic after treatment, and the patient's family was counseled regarding the removal of naphthalene mothballs from the home.

## 3. Discussion

Mothballs historically contained camphor but are currently more commonly comprised paradichlorobenzene or naphthalene [1]. Naphthalene-containing mothballs vary in weight and can contain up to 5 g of naphthalene [1, 5]. The metabolites of naphthalene include compounds such as alpha-naphthol which can cause oxidative stress leading to methemoglobinemia [1, 6]. This stress can also cause direct injury to red blood cells, resulting in hemolysis and anemia [1, 7]. Patients diagnosed with G6PD deficiency are especially susceptible to the development of hemolytic anemia when exposed to naphthalene [2, 8–10].

Clinical manifestations of naphthalene toxicity include nausea, vomiting, diarrhea, abdominal pain, increased creatinine, increased blood urea nitrogen, jaundice, elevated liver enzymes, hemoglobinuria, fever, headache, and altered mental status [5–8]. After ingestion, naphthalene is readily absorbed and metabolized by cytochrome P450 oxidation and later excreted in the urine as mercapturic acids, methylthio derivatives, and glucuronide conjugates [7]. Naphthol-alpha is the most potent metabolite of naphthalene, which causes hemolysis and severe anemia as well as Heinz bodies formation [7]. Methemoglobinemia occurs secondary to oxidation of hemoglobin to methemoglobin by naphthalene and its metabolites [4].

The toxic effect of naphthalene is due to an enhanced production of free oxygen radicals, resulting in lipid peroxidation and deoxyribonucleic acid damage [5, 6, 8]. Hemolysis occurs through either hemoglobin or cell membrane effects, particularly in patients with a low tolerance to oxidative stress, like G6PD deficiency [5, 8]. G6PD is essential in red cell metabolism through the pentose phosphate pathway, providing protection against oxidative stress on the cell [8]. G6PD deficiency is an X-linked recessive disorder, with male predominance, and has an incidence of about 400 million individuals globally. G6PD deficiency is the second most common human enzyme defect. Triggers of hemolysis in G6PD deficiency include infection, fava beans, and some antimalarial medications. G6PD deficient patients have decreased resistance to oxidative stress with a decreased production of the reduced form of nicotinamide adenine dinucleotide phosphate [8].

Treatment of naphthalene toxicity is supportive, with methylene blue as the antidote for cases of methemoglobinemia and blood transfusion for patients with hemolytic anemia [6]. Exchange transfusion can be performed in the setting of methemoglobinemia in G6PD deficiency due to the risk of induced hemolysis with methylene blue [6]. In mild cases, if the offending agent is removed, methemoglobin may return to normal hemoglobin within a few days [8]. Ascorbic acid and *N*-acetylcysteine have been studied for their purported antioxidant effects after naphthalene-induced oxidative stress, but results are inconclusive [6, 10, 11].

This case describes the effect of oxidative stress and subsequent hemolytic anemia secondary to ingestion of naphthalene-containing mothballs in a patient diagnosed with G6PD deficiency. The patient was successfully treated with blood transfusion and supportive care. On outpatient follow-up two months later, the hemoglobin and hematocrit were within normal limits at 13.0 g/dL and 39.5%, respectively. The patient's parents were educated regarding the avoidance of foods and substances that may trigger hemolysis in G6PD deficiency.

In conclusion, this case serves as a reminder that mothballs, a ubiquitous household item, can be dangerous when accessible to vulnerable children. Extreme care and caution should be taken to secure these products and prevent ingestion.
